# Functional-Network-Based Gene Set Analysis Using Gene-Ontology

**DOI:** 10.1371/journal.pone.0055635

**Published:** 2013-02-13

**Authors:** Billy Chang, Rafal Kustra, Weidong Tian

**Affiliations:** 1 State Key Laboratory of Genetic Engineering, Institute of Biostatistics, School of Life Sciences, Fudan University, Shanghai, P.R. China; 2 Dalla Lana School of Public Health, Division of Biostatistics, University of Toronto, Toronto, Ontario, Canada; National Taiwan University, Taiwan

## Abstract

To account for the functional non-equivalence among a set of genes within a biological pathway when performing gene set analysis, we introduce GOGANPA, a network-based gene set analysis method, which up-weights genes with functions relevant to the gene set of interest. The genes are weighted according to its degree within a genome-scale functional network constructed using the functional annotations available from the gene ontology database. By benchmarking GOGANPA using a well-studied P53 data set and three breast cancer data sets, we will demonstrate the power and reproducibility of our proposed method over traditional unweighted approaches and a competing network-based approach that involves a complex integrated network. GOGANPA’s sole reliance on gene ontology further allows GOGANPA to be widely applicable to the analysis of any gene-ontology-annotated genome.

## Introduction

Microarray-based case-control studies often begin by performing statistical differential expression analysis, and result in a list of significantly differentially expressed genes. The interpretation of such results often amounts to analyzing whether certain biological functions are enriched within the genes inside the gene list. For example, gene set over-representation analysis and its variants are popular approaches for downstream analysis upon differential expression analysis. Interested readers are referred to [1] and [2] for an overview of various gene set over-representation analysis methodologies.

An alternative approach, commonly termed gene-set-analysis (GSA) and initiated by [3], performs statistical differential analysis based on summary test-statistics evaluated using gene expression measurements of all the genes within pre-defined gene sets. Specifically, the null hypothesis of GSA is that genes belonging to a pathway are not collectively differentially expressed between two phenotype groups. One characteristic of GSA, as compared to more standard gene-wise approaches, is that if the subsets are chosen based on relevant biological knowledge, GSA may lead to more powerful tests by borrowing information across functionally similar genes. It can also lead to clearer interpretation by suggesting some biological features, rather than individual genes, that appear significant to the phenotype being studied. Variants of GSA, such as those proposed by [4] and [5], basically differ from each other by the construction of the test statistic and the choice of the null distribution.

The introduction of GSA is revolutionary, as it allows convenient interpretation of biological results, and enjoys higher power due to reasons described previously. With the steadily growing amount of information regarding functional groupings of genes from databases such as the Kyoto Encyclopedia of Genes and Genomes (KEGG) [6], Biocarta, Reactome [7], and MSigDB [3] to facilitate the convenient usage of GSA, GSA is now a mainstay technique for statistical analysis of gene expression data, either exploratory or confirmatory.

Classical GSA approaches, however, treat all genes within a gene set equally. Cognizant of the fact that gene sets are typically defined by the genes within a biological pathway, and that a pathway’s functions are induced by a group of genes in concert, the importance of genes with functions central to the pathway’s functionality should be emphasized; while a collection of differentially expressed genes can imply the significance of a pathway, a small set of differentially expressed gene can also imply the significance of a pathway if they are functionally crucial to the pathway of interest. Ignoring different functional classes of genes within a pathway may limits classical GSA’s its interpretability and biological relevance in application.

This problem has not been properly addressed until recently, when the functional non-equivalence among pathway genes are adjusted by exploiting the curated network topology of the pathway’s gene network available from various databases. For example, [8] and [9] consider weighting the importance of a gene based on how it is regulated by its direct upstream genes within the pathway network, while [10] weight genes according to their network distances from their neighbouring genes, and [11] further consider the genes’ distances from the terminal nodes of a pathway. However, possibly except for [10], all the above approaches require well-curated information regarding the pathway dynamics (e.g. induction and repression relationships for [8], [9], and the locations of terminal pathway genes for [11]), and hence are not applicable to more general gene sets without detailed network topological information.

In lights of the above issues, GANPA [12] attempts to integrate functional-linkages information among genes into the GSA framework by considering an integrated global gene network using a gene co-expression network, a protein-protein interaction (PPI) network, and a gene ontology (GO) based functional-linkage network. While previous approaches utilize the curated pathway network from various databases, GANPA instead considers the subnetwork of the global network, consisting only of the pathway’s genes, as the pathway network.

Although the utilization of the global network has eliminated the needs for potentially erroneously curated network topological information, the limited availability of PPI information for certain organisms limits GANPA’s applicability on certain, particularly non-modelled, organisms. Further, when constructing the GO-based functional-linkage network, GANPA ignores the semantic similarity between GO functions, and will link two genes only if they share certain specific biological functions, hence limiting the reliability and coverage of the global gene network.

In this article, we present GOGANPA, a Gene-Ontology and Gene Association Network-based Pathway Analysis tool. In GOGANPA, we construct a functional network by thresholding a gene-gene similarity matrix based on the Resnik similarity [13], which can account for the semantic similarities between various GO terms during network construction. Furthermore, GOGANPA does not require gene co-expression network and PPI network information; GOGANPA’s sole reliance on GO annotations allows GOGANPA to be applied to any GO-annotated genome, thus providing a more general network-based GSA framework, comparing to other network-based GSA approaches which require curated network information which are limited in availability.

## Materials and Methods

**Figure 1:**
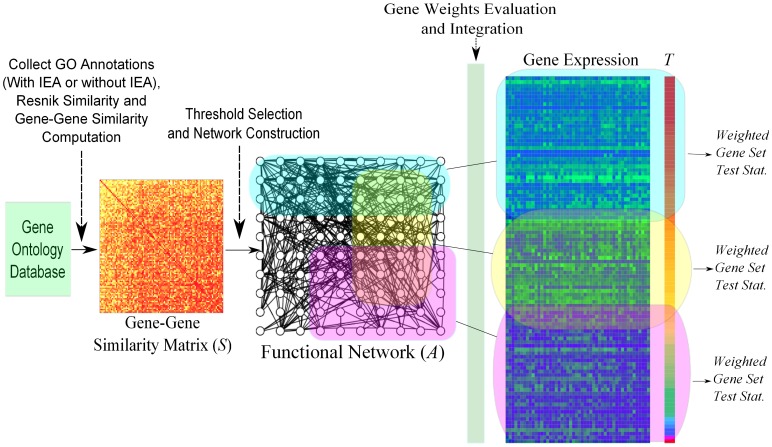
Overview of GOGANPA. GOGANPA transforms a GO similarity matrix into a gene network. Gene weights are then evaluated for each pathway (represented by transparent coloured boxes), and the weights are integrated into the gene expression data to evaluate the test statistics 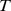 and weighted pathway test statistics.

Here we assume our data consists of 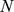 genes 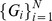, with their expressions measured across 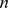 subjects. Further, we have 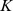 sets of gene sets 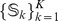, each representing the set of gene indices for the genes within a pathway, i.e. 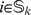 if the 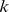
th pathway contains 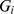. Our method for network-based GSA involves the following steps (Figure 1):

1. Compute the Resnik similarity for all pairs of genes in 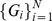.
2. Create a functional gene network by using the similarities obtained from step 1.
3. Compute a weight for each gene in each gene set using the information obtained from the network from step 2.
4. Incorporate the weights from step 3 into the GSA test statistic, and perform weighted GSA.

### Resnik Similarity

The first step of GOGANPA is to create a genome-wide functional similarity network. This will be achieved by considering the Resnik functional similarity between each pair of genes within the genome of interest. We will begin by briefly over-viewing the Resnik similarity, a measure of similarity between two GO terms. For complete details, please consult [13].

For every GO term 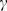, a specificity measure 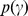 is first assigned to each GO term based on its number of annotated gene products. The Resnik similarity 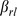 for two GO terms 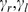 is then defined by:
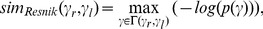
(1)where 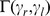 is the set of all common co-ancestors of 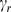 and 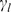 within the GO hierarchy.

For a pair of genes 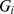 and 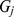, one first identifies 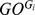 and 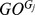, the set of GO-terms associated with gene 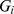 and gene 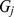 respectively. Assuming there are 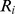 and 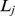 GO terms associated with gene 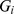 and 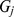 respectively, the Resnik similarities for the 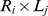 pairs of GO terms between 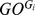 and 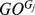 are then evaluated:
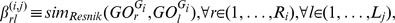
where 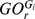 denotes the 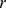
th annotated GO term for gene 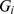, and 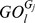 is defined similarly. A measure of functional similarity between gene 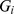 and 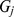 can then be defined as:



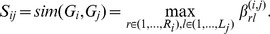
(2)Other similarity measures besides the Resnik measure (equation 1) are also available in the literature. Instead of using the maximum operator as in equation 2, the similarity between two genes can also be defined by combining the set of 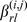 in alternative ways. See [14] for an overview of such alternatives.

The Resnik similarity is unbounded above. For ease of manipulation, in this article we will normalize each 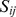 by its maximal entry:
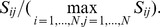



In the following sections, 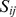 will correspond to the normalized Resnik similarity measure by default, and 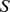 will represent the similarity matrix, with its 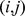
th entry equalling 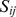.

In the GO database, functions are annotated to various genes in different manners. While certain annotations have been manually confirmed by curators, most annotations, with evidence code “IEA”, are computationally annotated based on homologs information, and have not been manually confirmed.

A similarity matrix constructed using non-IEA annotations may be more accurate due to the high quality, manually curated annotations used. Yet it may be less informative, as currently-available manually curated annotations are far from being complete. Although a similarity matrix constructed using all annotations (including those with evidence code IEA) may be noisier due to the low-quality annotations, the high coverage of gene functional annotations can result in a more informative network. In this article, we will explore both networks’ performances for network-based-GSA. We term GOGANPA the network-based-GSA method where the network is constructed without using IEA annotations, and we term GOGANPA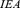 the method which utilizes the network constructed using both non-IEA and IEA annotations. More details regarding the annotations used will be presented in the “Data and other implementation details” section below.

### Similarity Transformation

We will now describe how to obtain a genome-wide functional network based on the similarity matrix 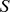 obtained above. A gene network is represented by two sets 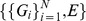, where each gene 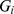 is a node, and 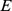 represents a set of gene-pairs, where 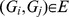 if gene 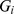 and gene 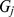 are connected by an edge. A gene network can be succinctly encoded by an adjacency matrix 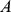, where 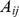, the 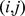
th entry of 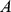, is 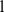 if gene 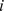 and gene 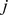 are linked by an edge, and 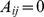 otherwise. We will not consider self-edges, and hence we set 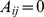 if 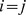.

To obtain an adjacency matrix, we threshold the similarity matrix 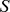:
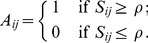
(3)


In other words, a pair of genes will be connected if their similarity lies above a certain threshold 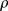 [15].

To determine an appropriate threshold 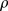 in (3), we will employ the scale-free-topology criterion for threshold selection [15]. Briefly, the network connectivity 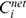 of a gene 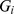 is defined as the number of genes connected to 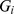 by an edge within the whole functional network. That is, 
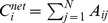
. Many past studies in gene networks suggest that the connectivities of all the nodes inside a network should follow a power-law distribution [16], i.e.
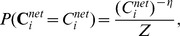
where 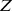 is the normalizing constant for the power-law distribution, 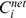 is the realization of the random variable 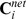, and 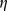 is a positive constant.

Based on this idea, [15] suggests a linear regression based goodness-of-fit test, testing how the observed network connectivity distribution fit against a power-law distribution. Briefly, by taking 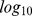 on both sides of the above equation, one obtains a linear relation:
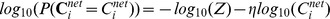



One may now divide the range of 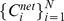 into, say, 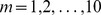 bins of equal lengths, and assign each 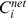 to the bins according to their values. Let 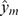 be the proportion of 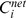
’s falling into the 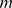
th bin, and 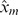 be the mean of the 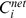 values inside the 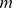
th bin. Treating 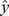 as an estimate of 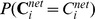, and considering the linear relation between 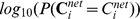 and 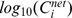, one can fit an ordinary least square regression model with predictors 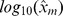 and responses 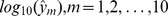. The typical goodness-of-fit measures for linear regression, 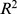, can then be used as a goodness-of-fit measure for 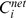 against the power-law distribution.

As such, one can fit a series of linear regression models, and obtain the corresponding series of 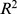, for a range of 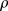. The 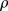 which achieves the maximum 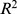 will be used for downstream analysis (Figure S1). For complete details for the above 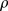 selection scheme, please consult [15].

### Gene Weights Evaluation

Upon obtaining the adjacency matrix 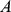 from the previous section, we are now ready to evaluate the gene weights for weighted-GSA, where the gene weights will reflect the importances for their respective genes within different gene sets. Similar to [12], GOGANPA construct gene weights based on a gene’s degree within a pathway, adjusted for its degree within the global network.

Along with the network connectivity 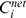 defined above, the pathway connectivity 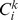 of gene 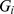 is defined as the number of genes connected to 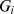 by an edge within the subgraph of the full genome-wide functional network, consisting only of the genes inside the 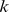
th gene set 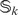. I.e. 
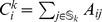
. It is worth noting that, as 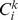 are defined based on a sub-network of the full functional network, 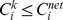 always.

Now, if gene 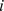 is significantly functionally associated to the genes inside 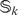, then most of gene 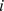
’s edges will be preferentially connected to genes inside 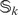, and to a significantly lesser extent, be connected to genes outside 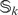. We will measure this significance using the hypergeometric distribution, as argued below.

If gene 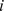 is not functionally associated with genes inside 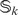, then among the other 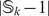 genes, the number of them gene 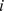 will be connected to will have a hypergeometric distribution with parameters 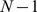, 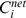, and 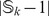. To see this, just imagine that all genes, except gene 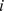, inside the full functional network are balls in an Urn. The ones connected to gene 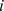 are the 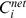 white balls, and the rest are black. If we randomly select 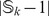 balls, the number of selected genes which are connected to gene 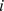 (i.e. the number of white balls) will follow a hypergeometric distribution.

Hence, if 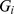 has no specific functional association with the genes in 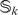, then the density function of the hypergeometric distribution provides:
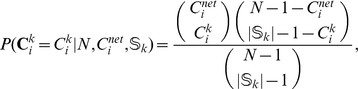
where 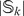 denotes the number of genes inside 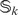. Under this distribution, the expected value of 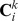 is:



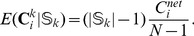
(4)The gene weight, 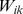, measuring the importance of 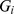 with respect to pathway 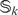, is defined in the following two steps:
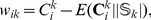
(5)

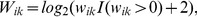
(6)where 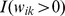 is the indicator function, equalling 1 if 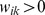 and 0 otherwise. As most genes are not functionally crucial for most pathways, the distribution of 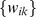 will be right-skewed. A log-transform is therefore applied to reduce the importances of those genes with unusually high 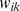, while allowing those genes with 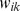 around the median to be more distinguishable from each other.

When the observed 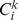 is smaller than 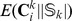 (for example, when 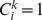 and 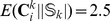), gene 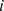 is potentially non-central to the 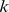 gene set. In this case, 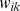 will be negative (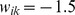 following from the example above). However, as most gene sets will only have a few numbers of crucial genes, we do not want to lose the potential information available from the non-central genes by under-weighting them. Hence, we reset negative 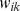 to 0 by using the thresholding function 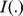 in equation 6. Such negative 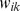 will then lead to 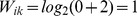. By setting weights for non-central genes to 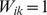, we will not lose their information when performing the downstream tests of significance, yet their contribution will not be emphasized.

**Figure 2:**
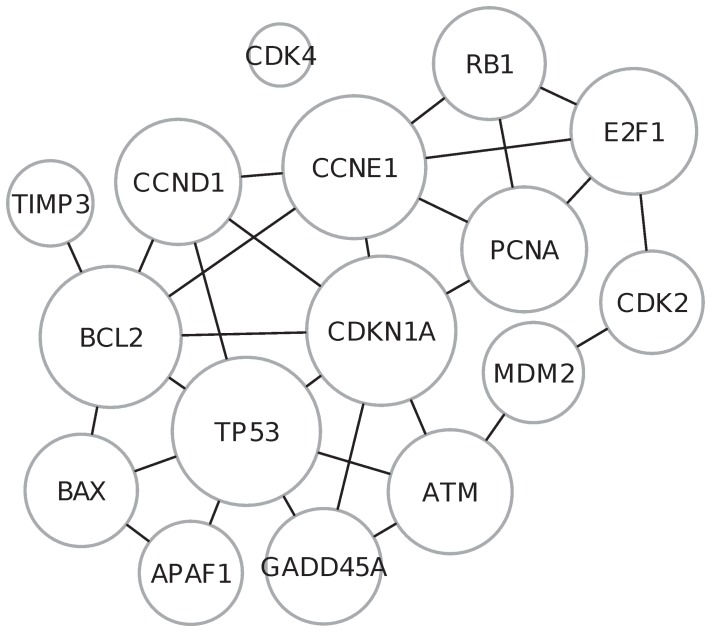
P53 Pathway. Node sizes correspond to the gene weights evaluated by GOGANPA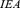. The functional-centrality of the *TP53* gene is highlighted by being assigned a high weight. The *CDKN1A* gene, a gene responsible for cell-cycle regulation and DNA-damage response, also receives a high weight due to its functional significance within the P53 Pathway.

A gene with a large weight for a pathway implies that the gene plays a function central to the pathway of interest. For example, in the P53 Pathway (Figure 2), a pathway describing how the p53 transcription factor controls cell cycle in the presence of damaged DNA, the central role of the *TP53* gene is highlighted by a high weight being assigned to it by the above weighting scheme. The *CDKN1A* gene, a gene responsible for cell-cycle regulation and DNA-damage response, also receives a high weight due to its functional significance within the P53 Pathway.

#### Multi-subunit protein correction

**Figure 3:**
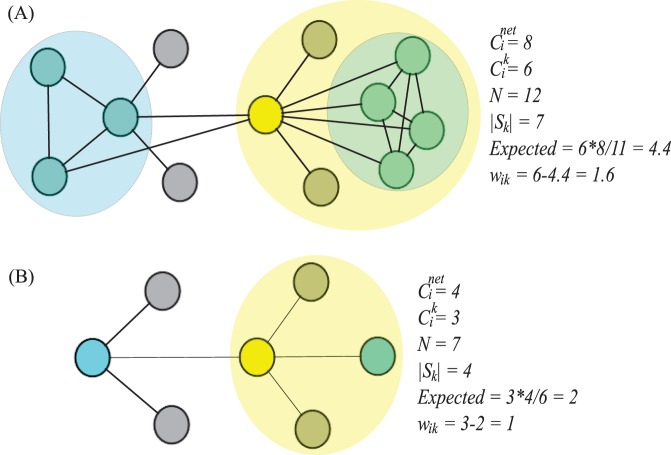
Illustrating MSP-correction. (A) The gene-of-interest (yellow node) is connected to certain single-protein-coding genes and two groups of MSP-coding genes (inside blue shades). The presence of MSP-coding genes inflates both 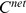 and 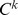 for the gene-of-interest inside the pathway shaded in yellow. (B) Upon collapsing the MSP-coding gene groups into single units, both 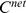 and 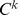 are reduced at the protein level.

Certain genes in the human genome, e.g. the ANAPC family of genes, are responsible for encoding the subunits of a multi-subunit protein (MSP). The ignorance of the existence of MSP-coding genes may lead to the “over-counting problem” [12], where the MSP-coding genes may unnecessarily inflate the weights of the genes connected to such MSP-coding genes, and consequently masking the importance of the other genes within a gene set. Figure 3A presents a toy gene network with two groups of MSP-coding genes. The gene of interest (yellow node) will have a high network connectivity and pathway connectivity due to the existence of the MSP-coding genes, thus inflating the weight for this gene-of-interest. Cognizant of the fact that such MSP-coding genes will share similar functions as they encode the same gene product, such genes can be collapsed to a single unit when evaluating connectivities, as demonstrated in Figure 3B.

To correct the MSP problem, we employ the approach described in [12]; we simply collapse MSP-coding genes into a single unit prior to connectivity evaluation. MSP-corrected gene weights are then simply evaluated by (4–6) on the collapsed gene-network.

### Weighted Gene Set Enrichment Analysis

In standard GSA, one first obtains a set of test-statistics 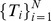 for each gene 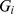 (e.g. the test statistics for the 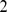-sample 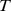-test, or the Kolmogorov-Smirnov-like statistics pioneered by [3]). Then a summary statistic for a gene set 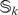 is computed by applying a function on 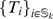. In this article we will employ the mean-absolute statistic:
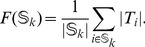
(7)


To incorporate the weights obtained in the previous section, we modify the above equation by:
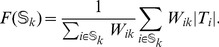
(8)


Weighted-GSA then simply follows the typical permutation procedure: we create 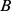 copies of our original expression data, but with the phenotype class labels randomly permuted. We then obtain 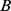 sets of test-statistics 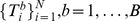, and subsequently:
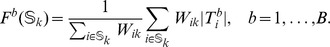



A permutation 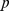-value for 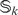 can then be evaluated as:
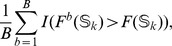
where 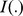 is the identity function, equalling 1 if the condition inside 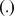 is true, and 0 otherwise. To correct for multiple-testing, we consider controlling the false-discovery rate (FDR) [17] and investigate the resulting sets of 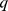-values [18].

We further consider a normalized test-statistics, 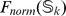, proposed in [12], which is simply the original gene set test statistic (7, 8), but subtracted by the median and divided the standard deviation of all the gene sets’ permuted test-statistic values, i.e.:
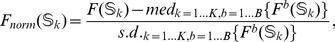
(9)where 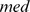 and 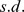 are the median and standard deviation operator. 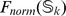 provides a measure of effect size of the correlation between 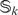 and the phenotype of interest, while the normalization allows the test-statistics to be compared across pathways with different sizes.

In practice, a measure of statistical significance (e.g. 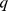-value) and a measure of effect size (e.g. 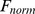) are both important for decision making; a significant gene set with a large effect size is potentially more biologically interesting than a significant gene set with a small effect size. Therefore, besides the 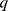-values, 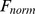 can provide another way to assess the gene sets’ biological relevance. In particular, in the presence of a huge number of significant gene sets, one can utilize the 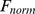 scores to prioritize the biological relevance of such significant gene sets. We have employed this ranking procedure in two of the three experiments presented below.

### Data and Other Implementation Details

For the choice of the global gene network, GANPA [12] utilizes an integrated network, where two genes are linked together if either they regularly co-express, they translate interacting proteins, or they share certain specific GO functions. GOGANPA, on the other hand, utilizes the functional network constructed as described in section 2.1 and 2.2. We obtained GO annotations from the R Bioconductor package *org.Hs.eg.db*, version 2.6.4. GO annotations with evidence code “ND” (no biological data available) are excluded for functional network construction. As mentioned in the Resnik Similarity section above, GOGANPA will not use electronically inferred annotations (GO evidence code “IEA”) when building the functional network, and we will consider a variant of GOGANPA, termed GOGANPA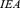, which will further utilize IEA annotations when calculating the pair-wise gene-gene similarities.

To construct the functional network for the various GOGANPA variants, we first obtain pairwise gene similarity matrix 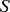 using the R package *csbl.go* [19], version 1.3.6, available from the package website. We only use GO Biological Process functional annotations for similarity calculation. To obtain the adjacency matrix 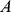, the power-law goodness-of-fit test described above has chosen thresholds 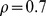 and 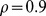 for GOGANPA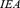 and GOGANPA, respectively (Figure S1). This will result in two networks with 6,456 genes (143,697 gene-pairs) and 1,060 genes (751 gene-pairs) for GOGANPA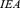 and GOGANPA respectively.

Unless stated otherwise, in this article, GOGANPA will refer to the weighted-GSA method with weights derived from the network constructed without using “IEA” and “ND” annotations, and with 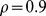. On the other hand, GOGANPA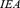 will refer to the weighted-GSA method with weights derived from the network constructed using all annotations (including “IEA” annotation, but excluding “ND” annotations), and with 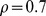.

We will compare five gene set analysis methods: the Kolmogorov-Smirnov based method (KS) [3], the unweighted method using the absolute mean test statistic (7) (absM) [5], and the three weighted-GSA methods (8) with weights evaluated according to the GANPA, GOGANPA, and GOGANPA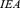 pipelines, which differ from each other by the gene network involved. For KS, we use the software downloaded from the website of [3]. For absM and GANPA, we use the R-package GANPA available on the CRAN R-repository.

The p53 data set was downloaded from the website of [3]. KS was applied to the data as downloaded, while for the other methods, the data was preprocessed as described in [12] before being analyzed by absM, GANPA, GOGANPA and GOGANPA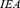. The three breast cancer data sets (GSE3744, GSE10780, and GSE14548) and the asthma data set (GSE18965) were downloaded from the NCBI Gene Expression Omnibus database, and preprocessed as in [12]. The 522 functional gene sets used in the p53 analysis, and the 833 gene sets used in the breast cancer and asthma studies were downloaded from MSigDB [3].

Genes inside the data being analyzed, but not inside the gene-network, will be assigned the basic weight 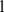 in the three network-based GSA methods.

Unless stated otherwise, the FDR thresholds are chosen as those employed in [12], whenever appropriate, for consistency with previously published results. As there is currently no standard FDR threshold established by the research community, the choice of the FDR threshold is somewhat arbitrary. In practice, increasing the FDR/ranking threshold will result in more significant gene sets, yet the number of false-discoveries will also increase. Users are therefore suggested to choose this threshold appropriately, according to the number of false-discoveries they can tolerate.

Principled methods for power or accuracy analysis for GSA methods, such as sensitivity/specificity analysis or cross-validation, require a reference “ground truth set” of positive and negative gene sets, i.e. gene sets known to be related, and known not to be related, respectively, to the phenotype-of-interest [9]. Currently, a lack of such ground truth set of gene sets has made principled evaluation of GSA methods impossible; while some gene sets have been documented in the literature to be correlated to certain phenotypes, the documentation is far from being complete, thus introducing difficulties in establishing a set of positive gene sets. Furthermore, establishing non-existence of relationship between gene sets and phenotypes is experimentally difficult, and is generally non-interesting to the scientific community. Documentations of such negative results will therefore be even rarer, constituting difficulties in creating a set of negative gene sets. While simulations provide an alternative approach for power analysis, such simulations are only meaningful when the data collection scheme is carefully designed according to the stochastic model behind the chosen hypothesis test, and can shed little light on the power of GSA methods in our exploratory analysis setting. Therefore, as a guide to the compared methods’ efficacy and validity in the absence of a “ground-truth set”, we will check whether the gene sets deemed significant by our methods are consistent with the published results from the literature, as well as a reproducibility analysis [9] described in the “Breast Cancer Data” section below.

An R-package, GOGANPA, which implements our proposed method, is available at the CRAN R-repository.

## Results

### p53 Status in Cancer Cell Lines

The p53 dataset has been widely used for validating pathway analysis algorithms. The data set contains 17 p53-wild-type (WT) and 33 p53-mutated (MUT) cancer cell lines, with their gene expression measured across 10,100 genes. We limit our analysis to gene sets with size between 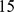 and 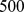, leaving us with 308 gene sets from the original 522 gene sets for analysis. 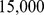 permutations are performed for each method being compared. Controlling FDR at 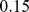, we consider gene sets with 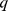-value 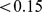 as significant.

The results are presented in Table 1. KS and absM can only identify, respectively, five and six pathways as significant, while GANPA has identified 10 significant pathways, and GOGANPA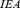 has identified 16 pathways as significant. It’s reassuring, furthermore, that GOGANPA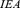 has discovered all 10 pathways deemed significant by GANPA, suggesting its solid improvement over GANPA. GOGANPA, without IEA annotations, has only discovered 12 significant pathways, suggesting that IEA annotations can provide further insights into the pathways’ correlations with the phenotype of interest.

**Table 1:** p53 Data – Results.

Pathway	KS	absM	GANPA	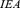 GOGANPA	GOGANPA
p53 hypoxia pathway	**0.001**	**0.015**	**0.01**	**0.005**	**0.015**
hsp27 pathway	**0.002**	**0.033**	**0.09**	**0.029**	**0.033**
p53 pathway	**0.006**	**0.015**	**0**	**0**	**0.01**
p53 up	**0.01**	**0.015**	**0**	**0**	**0**
radiation sensitivity	**0.064**	**0.015**	**0**	**0**	**0.014**
ck1 pathway	0.474	0.178	0.157	**0.139**	**0.145**
bad pathway	0.507	**0.079**	**0.125**	**0.049**	**0.067**
p53 signalling	0.517	0.22	**0.125**	**0.041**	**0.209**
st dictyostelium	0.788	0.178	0.157	**0.106**	**0.145**
G2 pathway	0.8	0.22	0.198	**0.106**	**0.212**
bcl2 family and reg network	0.828	0.22	**0.125**	**0.08**	**0.141**
DNA damage signalling	0.862	0.178	0.198	0.2	**0.141**
ceramide pathway	0.874	0.189	0.157	**0.038**	**0.177**
mitochondria pathway	0.881	0.178	**0.127**	**0.106**	**0.044**
cell cycle pathway	0.899	0.178	0.151	**0.107**	**0.145**
cell cycle arrest	0.958	0.22	0.157	**0.095**	**0.209**
cell cycle regulator	0.969	0.178	**0.125**	**0.078**	**0.152**
Total Significant Pathways	5	6	10	16	12

Controlling FDR at 0.15, the 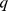-values obtained by each method for the pathways deemed significant by at least one of the five methods are listed, with 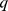-values 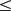 0.15 boldfaced. The absM method can only identify six pathways, while GANPA can identify four more. Compared to GANPA, GOGANPA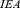 can discover six more pathways, while discovering all the pathways deemed significant by GANPA. Abbreviation: st dictyostelium: st dictyostelium discoideum camp chemotaxis pathway.

Among the pathways considered significant by GOGANPA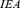 but not by the unweighted methods or GANPA, a number of them are well-known to be related to p53 functions. These includes the mitochondria pathway, the BCL2 network, and the ceramide pathway, which are related to apoptosis [20], [21]. p53 functions in cell cycle are also reflected by the significance of the cell cycle, cell cycle arrest, and cell cycle regulator pathways [22], [23]. p53-dependent actions of the G2 pathway is also well documented in the literature [24].

As discussed in [12], the HSP27 Pathway, known to be functionally related to p53 functions [25], is somehow given a higher 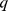-value by GANPA (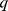-value = 0.09) compared to that by the absM (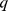-value = 0.033). It is worth-noting that GOGANPA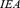 can assign the HSP27 Pathway a lower 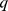-value (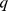-value = 0.029), which is more biologically relevant.

**Figure 4:**
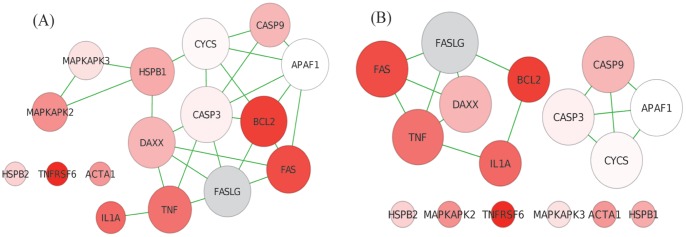
p53 Data - HSP27 Pathway. Deeper colour represents stronger differential expression (i.e. higher 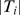). Grey nodes represent genes with missing expression measurements. Node sizes correspond to the gene weights evaluated by GANPA (A) and GOGANPA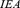 (B). Comparing to GANPA, while GOGANPA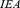 has down-weighted the differentially expressed *BCL2* gene and *MAPKAPK2* gene, it has up-weighted the differentially expressed *FAS, TNF*, and *IL1A* genes, and has hence produced a higher pathway test statistic and a smaller 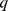-value for the HSP27 Pathway.

To obtain better insights into GANPA’s and GOGANPA
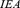
’s results, Figure 4 presents the HSP27 Pathway network, indicated with its genes’ test statistics for differential expression (i.e. 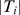) and their gene weights evaluated by GANPA and GOGANPA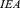. Comparing to GANPA, although GOGANPA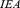 has down-weighted the highly-differentially-expressed *BCL2* and *MAPKAPK2* gene, GOGANPA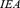 has up-weighted the highly-differentially-expressed *FAS, TNF, and IL1A* genes, resulting in a smaller 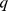-value for the HSP27 Pathway. Note that the highly-differentially-expressed *TNFRSF6* gene is heavily down-weighted by both GANPA and GOGANPA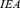, a potential reason why absM can somehow provide the HSP27 Pathway a low 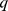-value.

**Figure 5:**
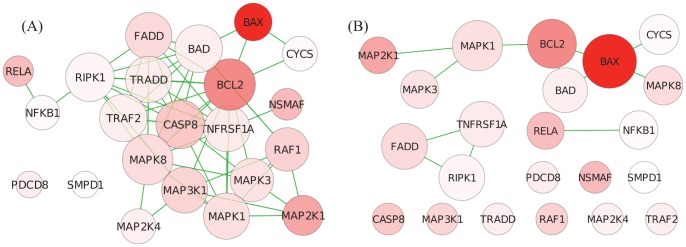
p53 Data - Ceramide Pathway. See caption of Figure 4 for descriptions. The highly differentially expressed *BAX* gene, considered less important by GANPA (A), has been strongly up-weighted by GOGANPA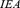 (B), allowing GOGANPA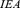 to discover the ceramide pathway’s significance.

For a clearer comparison, we further investigate the Ceramide Pathway (Figure 5), whose functions are regulated by p53 [21], and is deemed significant by GOGANPA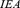 (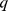-value = 0.038) but not by the other four methods. The *BAX* gene, which clearly stands out as a highly-differentially-expressed gene inside the Ceramide Pathway, is significantly up-weighted by GOGANPA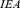, but significantly down-weighted by GANPA. Unlike the HSP27 Pathway, which contains a fair amount of highly-differentially-expressed genes, the significance of the Ceramide Pathway can only be discovered if the singly differentially-expressed *BAX* gene is up-weighted, as done by GOGANPA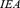, but not by GANPA.

Besides the values of 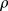 chosen by the scale-free-fitness test, we have also explored how GOGANPA and GOGANPA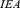 perform under 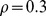 and 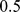 (Table S1). We find that GOGANPA with 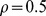 can obtain 17 significant pathways, one more compared to that of GOGANPA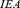 with 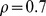 (the 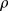 chosen by the scale-free-fitness test). This suggests that, without IEA annotations, GOGANPA can still achieve comparable performances compared to GOGANPA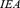, if a suitable 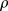 can be chosen appropriately.

To investigate how the results may vary under different 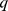-value threshold, we have also explored the results obtained under 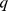-value threshold 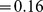. Under this new threshold, GOGANPA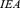 with 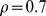 can identify 20 significant gene sets, the highest number of significant gene sets obtained among all methods being compared. It is worth noting that GANPA and GOGANPA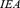 with 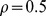 can identify significantly more pathways (17 and 19, respectively), compared to that when the 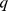-value threshold was set at 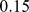 (Table S1).

### Breast Cancer Data

One of the main advantages of GSA is its robustness against independently repeated experimentation, possibly done with different platforms [3]. Due to limited sample sizes, the outcomes of single-gene differential expression analysis are often highly variable; experimental data of the same phenotypic nature, but collected by independent groups, often leads to different results. In GSA, the fact that a gene can “borrow information” from its neighbouring pathway genes through a pathway test-statistic can thus increase the stability and reproducibility of the analytic outcome. In this section, we will investigate the reproducibility of GOGANPA and GOGANPA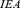. While we will still provide certain in-depth analysis of some pathways, the focus of this section is reproducibility, but not the interpretation of the results.

We analyzed three breast cancer data to identify the conserved significant pathways across the three different data sets. Among the 620 gene sets (with size between 15 and 500) and controlling FDR at 0.15, absM, GANPA, and the two GOGANPA variants have generated a huge amount of significant pathways (more than 600 in all three data sets). For the three breast cancer data sets, at 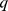-value threshold set at 0.15, KS can only discover 58 significant gene sets in the GSE14548 data set, and 0 significant gene sets in GSE3744 and GSE10780. The lack of significant gene sets discovered by KS precludes us to analyse the conservation ability of KS across the three breast cancer data sets, and we hence exclude KS from our analysis in this experiment.

To compare the various methods in the presence of a huge amount of significant pathways, we consider the normalized test statistics, 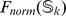 (9). For each method being compared, a pathway is considered conserved if its three normalized test-statistics, obtained from each of the three data sets, are ranked above 80.

As suggested in the analysis in [12], multi-subunit-protein (MSP) correction is employed in GANPA and the two GOGANPA variants (see the Methods and materials section for details regarding MSP-correction). 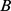 = 15,000 permutations are run for each method on each data set, and the results are presented in Table 2. While GANPA has conserved 14 pathways, GOGANPA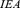 has obtained 17 conserved pathways, and hence has further outperformed GANPA in terms of reproducibility. GOGANPA, without IEA annotations, has apparently under-performed comparing to GANPA and GOGANPA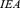 by conserving only 11 pathways.

**Table 2:** Breast Cancer Data – Results.

Database	Pathway	absM	GANPA	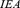 GOGANPA	GOGANPA
reactome	syn. di/tri-phosph.	**1,23,7**	**4,12,14**	**1,27,6**	**1,24,7**
reactome	metablism nts.	4,81,6	**6,54,7**	**3,56,10**	**4,78,10**
kegg	focal adhesion	**8,25,54**	**12,29,59**	**5,24,37**	**6,21,48**
kegg	pathways in cancer	**14,17,37**	**14,18,30**	**21,18,24**	**19,19,40**
biocarta	AGR pathway	**20,18,1**	**33,30,1**	**37,80,1**	**20,18,1**
kegg	melanoma	27,152,101	25,96,77	**17,69,35**	16,115,80
kegg	acute myeloid leukemia	**28,28,57**	**47,42,62**	**27,30,18**	**26,29,60**
kegg	pancreatic cancer	30,39,85	**34,36,48**	**36,67,39**	**34,39,76**
reactome	G2/M transition	38,30,90	**31,32,58**	34,15,108	36,30,89
kegg	prostate cancer	**39,12,12**	**37,19,8**	**62,19,2**	**45,5,9**
kegg	p53 signaling pathway	**40,9,24**	**30,5,60**	33,6,88	**39,9,22**
kegg	axon guidance	**48,8,11**	**61,9,4**	**51,8,5**	**50,10,11**
biocarta	PDGF pathway	50,96,114	21,58,81	**25,40,47**	64,114,125
reactome	cell cycle checkpoints	**55,22,80**	35,17,106	44,7,128	55,20,86
kegg	renal cell carcinoma	**71,55,10**	**93,55,10**	**60,72,12**	85,56,8
kegg	aldo. reg. Na reabs.	76,163,124	90,77,109	**58,78,71**	90,171,130
reactome	APC	**80,53,22**	**65,48,18**	**30,9,57**	82,51,20
kegg	reg. actin cyto.	84,87,71	77,61,84	65,90,53	**75,71,59**
reactome	down strm. sig. trans.	87,102,53	**53,60,54**	**56,51,25**	70,88,39
reactome	CDC20	92,113,15	81,111,12	**43,47,33**	91,111,15
biocarta	longevity pathway	109,154,87	73,112,55	**48,76,72**	108,149,87
kegg	glioma	111,151,77	**74,64,37**	83,98,27	104,144,66
Total Conserved Pathways		11	14	17	11

Pathways with normalized test-statistics ranked above 80 in all three data sets by at least one method are listed. The rankings of the pathway obtained from the three breast cancer data sets are recorded. Rankings above 80 across all three data sets are boldfaced. GOGANPA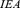 has identified the most number of conserved pathways across the three data sets. Abbreviation: syn. di/tri-phosph.: synthesis and interconversion of nucleotide di and triphosphates; metabolism nts.: metablism of nucleotides; aldo. reg. Na. reabs.: aldosterone regulated sodium reabsorption; APC: regulation of APC/C activators between G1/S and early anaphase; reg. actin cyto.: regulation of actin cytoskeleton; down strm. sig. trans.: down stream signal transduction; CDC20: Cdc20 Phospho-APC/C mediated degradation of Cyclin A.

**Figure 6:**
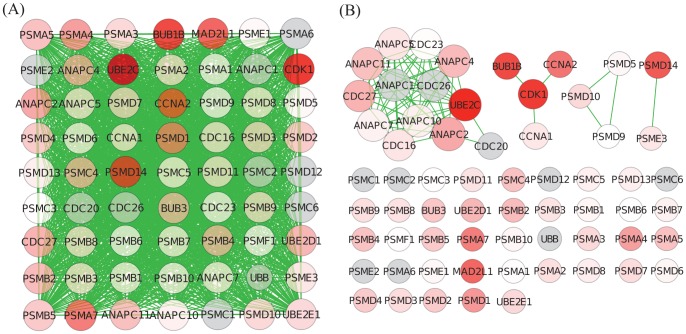
Breast Cancer Data: CDC20 Pathway. See caption of Figure 4 for descriptions. (A) In GANPA, a huge amount of co-expressing genes-pairs form a strongly connected network, and GANPA cannot distinguish the highly differential genes from the other less differentially expressed genes. (B) GOGANPA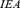, on the other hand, only considers functional relationships, and hence provides a much sparser network that highlights the importance of the highly differentially expressed *UBE2C* and *CDK1* genes.

We select the Cdc20:Phospho-APC/C Mediated Degradation Of Cyclin A (CDC20) Pathway, a pathway conserved across the three breast cancer data set only by GOGANPA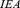, and investigate the gene weights and the test statistics for differential expression of the genes within this pathway in one of the three breast cancer data sets (Figure 6). According to the integrated network used in GANPA, the CDC20 Pathway, being a set of highly co-expressed genes, appears as an almost fully-connected network. The lack of variation in gene-weights has therefore disallowed GANPA to discover the significance of the CDC20 Pathway. GOGANPA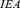, on the other hand, only considers GO-based functional similarity, and is able to provide a much sparser network for the CDC20 Pathway that highlights the importance of the highly-differentially expressed *UBE2C* and *CDK1* genes, leading to the discovery of the CDC20 Pathway’s significance.

We have further explored the conversation ability of GOGANPA and GOGANPA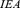 under 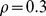 and 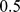 (Table S2). Upon comparison, GOGANPA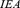 at 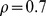, i.e. the 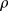 chosen by the scale-free-fitness test, still performs best by conserving 17 gene sets, followed by GANPA and GOGANPA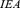 at 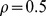 (14 pathways conserved by both methods).

### Asthma Data Analysis

We have seen from the above two analyses that the gene weights, as assigned by GANPA and GOGANPA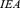, will have a significant impact on the results. To further explore the differences between GANPA’s and GOGANPA
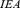
’s weights assignments and their potential impact on the results, we have further analysed a data set containing gene expression measurements from seven healthy and nine asthmatic children [26]. Following [12], multi-subunit correction was performed for GANPA, GOGANPA and GOGANPA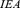 in this analysis and 10,000 permutations were performed for the permutation tests.

Among the 620 gene sets (with size between 15 and 500) being analysed, KS cannot deem any gene sets significant at FDR threshold 0.1, whilst the other 4 methods have obtained more than 100 significant gene sets at the same FDR threshold. We rank the significant gene sets by their normalized score 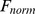, and present the top 10 gene sets, according to their 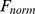 ranks, in Table 3.

**Table 3:** Asthma Data – Results.

Database	Pathway	absM	GANPA	GOGANPA	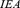 GOGANPA
kegg	renin angiotensin	4.83 (1)	4.16 (10)	4.37 (4)	4.1 (6)
biocarta	RAC1	4.67 (2)	4.23 (8)	4.67 (2)	4.39 (4)
reactome	carbohydrates	4.59 (3)	4.65 (1)	4.75 (1)	4.4 (3)
reactome	glucose transport	4.37 (4)	3.7 (22)	4.38 (3)	4.43 (2)
biocarta	ECM	4.37 (5)	3.73 (19)	4.37 (5)	3.83 (11)
reactome	pyruvate	4.29 (6)	3.6 (28)	4.29 (6)	3.49 (26)
biocarta	CTCF	4.26 (7)	4.38 (4)	4.19 (8)	4.22 (5)
reactome	basigin	4.19 (8)	4.31 (6)	4.19 (7)	3.31 (41)
reactome	telomere ends	4.16 (9)	3.71 (20)	4.08 (14)	3.62 (14)
kegg	glycosaminoglycan	4.13 (10)	3.67 (24)	4.13 (9)	3.92 (10)
reactome	glycolysis	4.13 (11)	4.55 (2)	4.13 (10)	3.46 (29)
reactome	bile acids/salts	4.09 (12)	4.47 (3)	4.09 (13)	3.96 (8)
kegg	pentose phosphate	3.58 (30)	4.32 (5)	4.1 (12)	3.62 (15)
reactome	gluconeogenesis	3.91 (16)	4.3 (7)	3.9 (17)	3.42 (32)
kegg	glycolysis gluc.	3.51 (35)	4.23 (9)	3.51 (34)	3.48 (27)
kegg	ARVC	4.05 (13)	3.39 (38)	4.12 (11)	3.95 (9)
biocarta	P53 hypoxia	3.6 (28)	4.08 (12)	3.6 (25)	4.67 (1)
biocarta	VEGF	3.65 (26)	3.7 (21)	3.57 (28)	3.97 (7)

The pathways’ 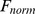 scores and rankings (in brackets) as scored and ranked by the four GSA methods are presented. All pathways presented have 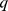-value 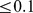, and have 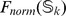 ranked within top 10 by at least one of the methods being compared. Abbreviations: carbohydrates: metabolism of carbohydrates; pyruvate: pyruvate metabolism and TCA cycle; basigin: basigin interactions; telomere ends: packaging of telomere ends; glycosaminoglycan: glycosaminoglycan degradation; bile acids/salts: metabolism of bile acids and bile salts; glycolysis gluc.: glycolysis gluconeogenesis; ARVC: arrhythmogenic right ventricular cardiomyopathy arvc.

**Figure 7:**
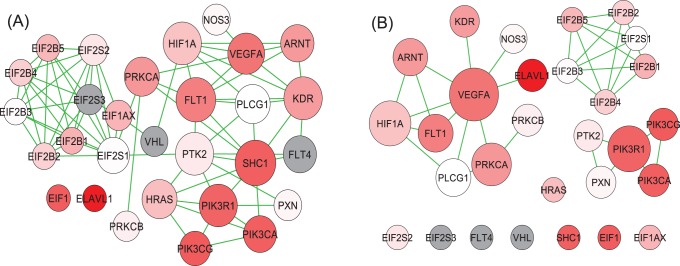
Asthma Data: VEGF Pathway. See caption of Figure 4 for descriptions. (A) The GANPA network. (B) The GOGANPA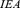 network.

A fair numbers of gene sets are ranked highly by all four methods being compared. For example, the renin angiotensin pathway, the RAC1 pathway, the carbohydrates pathway, and the CTCF pathway are ranked within top 10 by all four versions of GSA. On the other hand, GOGANPA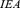 highly ranks the VEGF pathway, a pathway known to be related to asthma [27] (rank 7th), while GANPA ranks this pathway at 21st. Figure 7 shows the GANPA network (Figure 7A) and GOGANPA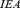 network (Figure 7B) for the VEGF pathway. The main difference between the two networks lie in their sparsity; the GANPA network is more connected, hence although many differentially-expressed genes have received high gene weights due to their high connectivities, their importances within the network cannot be emphasized due to the existence of other highly-connected and highly-weighted genes. The GOGANPA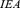 network, on the other hand, is much sparser, hence certain differentially-expressed genes, e.g. 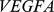 and 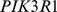, have obtained gene weights much higher than the other genes within the VEGF pathway. Furthermore, the highly differentially expressed 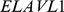 gene, being un-connected within the GANPA network, is assigned the basic weight 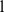 by GANPA, but has obtained a higher weight from the GOGANPA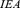 network due to its connection with the 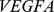 gene. Taken together, by highlighting the importances of certain differentially expressed genes, GOGANPA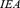 is able to provide the VEGF pathway a higher ranking than GANPA.

**Figure 8:**
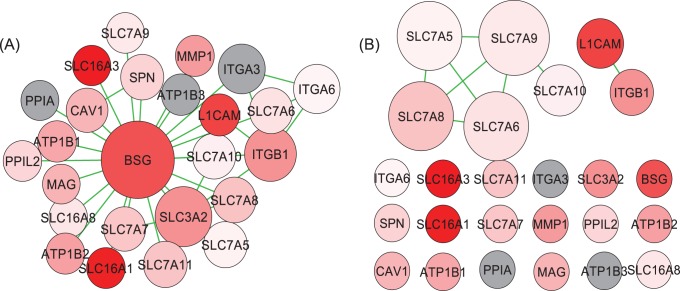
Asthma Data: Basigin Interaction Pathway. See caption of Figure 4 for descriptions. (A) The GANPA network. (B) The GOGANPA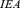 network.

On the other hand, the Basigin Interaction pathway is highly ranked by GANPA (rank 6), yet lowly ranked by GOGANPA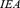 (rank 41). Figure 8 presents the GANPA network (Figure 8A) and the GOGANPA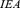 network (Figure 8B) for the Basigin Interaction pathway. For this particular pathway, GANPA can successfully emphasize the centrality of the 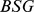 gene, while GOGANPA
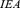
’s network is extremely sparse. Due to an under-informative network, GOGANPA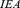 is not able to rank the Basigin Interaction pathway as high as that by GANPA.

We recall here that the GANPA network is a hybrid network constructed using PPI, gene co-expression, and functional linkage information. The GOGANPA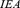 network, on the other hand, relies completely on functional linkage information obtained from the GO database. As a hybrid network, GANPA’s network will be denser, and will often be unable to distinguish the importance of certain pathway genes, as demonstrated in the VEGF network. In contrary, although GOGANPA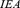 may be able to better-distinguish the functional importance of certain genes, the incompleteness of GO annotations may disallow GOGANPA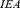 from providing informative pathway sub-networks, as illustrated in the above Basigin Interaction pathway example.

Nonetheless, our analysis here has demonstrated that both GANPA and GOGANPA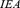 can have their unique strengths in identifying the significance of different pathways. Accounting for the fact that GOGANPA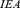 only requires functional annotations from GO, GOGANPA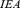 is necessarily simpler and more general than GANPA, a method which involves a significantly more complicated gene network.

## Discussion

Our methods differ from most other network-based GSA approaches in the following aspects: besides the gene-expression data, our methods only require GO annotations, while other methods require a combination of different network data sources, or information regarding network topology. Further, we consider using GO semantic similarities in our network construction step, hence allowing us to create a more informative GO functional network, comparing to the network obtained by naively identifying genes with shared GO functions.

The results of the p53 and breast cancer data analysis have demonstrated the superior power of GOGANPA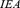 over GANPA and absM. The breast cancer data analysis has also demonstrated GOGANPA
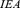
’s reproducibility across different data sets. Furthermore, the fact that GOGANPA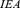 can significantly outperform GOGANPA signifies the importance of IEA annotations; although false annotations may exist within IEA annotations, the incorporation of IEA annotations allows genes without manually curated annotations to be considered during network construction, and will hence provide in a more comprehensive gene functional network for GSA, leading to the increased power of GOGANPA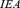 over GOGANPA.

The running-time of GOGANPA and its variants will depend on the sample size, the number of genes, the number of gene sets, and the number of permutations. For the p53 dataset, with 50 samples, 10,100 genes, and 522 gene sets, 15,000 permutations took GOGANPA and its variants approximately 9 minutes to complete on a laptop with an Intel Core i7, 1.90 GHz, 4MB L3 cache processor and 8 GB RAM. Significant speed-up can be achieved by reducing the number of permutations, but we recommend running no less than 10,000 permutations for accuracy and results stability.

At first glance, it may be counter-intuitive to believe that GOGANPA, which only utilizes GO annotations, can outperform GANPA, which involves a global network integrated from various data sources. However, when integrating PPI and gene co-expression networks into a GO functional network, as done in GANPA, one assumes that genes with interacting gene-products or genes being co-expressed are functionally related, without regards to the possibility that such gene-pairs may not necessarily be functionally related. In other words, GANPA inherently ignores the existence of falsely-linked gene-pairs within the integrated network. The analysis of the CDC20 Pathway (Figure 6), for example, suggests that integration of gene co-expression and PPI networks may produce highly-connected sub-networks, hence masking the importance of the regulatory genes within certain pathways. Although the gain in performance by GANPA over absM has demonstrated the usefulness of the integrated network, the superior performance of GOGANPA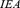, with a much smaller functional network compared to the integrated network used by GANPA, suggests that a high-quality functional network, constructed using well-curated and computationally predicted annotations, is far more valuable than a large, but noisy, integrated network.

The choice of the similarity threshold, 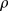, based on the scale-free-topology criterion may deserve more elaboration on its appropriateness. Many large-scale networks, such as gene-regulatory network and protein-protein interaction (PPI) network, have been documented in the literature to exhibit an approximate scale-free-topology (i.e. the degree distribution follows a power-law distribution) [16]. Though the scale-free-topology criterion for functional-linkage networks has not been studied to our knowledge, we argue that as co-expression and PPI are correlated to gene-gene functional similarity, particularly when the similarity is measure by the Resnik measure with the 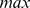 mixing strategy [28] (which we have employed in our paper), functional-linkage network will also be approximately scale-free, due to the scale-freeness of gene-regulatory and PPI networks.

We shall add a note of caution for the readers, that many small-scale networks will unlikely be scale-free. Also, the scale-free topology of a functional-network can be destroyed if it is constructed using a biased selection of genes [29]. This may occur when the experimenters are considering only a small selection of genes-of-interests for functional network construction, or if the organism being studied has insufficient functional annotations. The default network used in GOGANPA and GOGANPA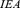 are genome-wide, and they hence will unlikely suffer from the issues discussed above.

In summary, we have introduced in this article GOGANPA and its variant GOGANPA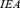, two GO-functional-network-based GSA methods. The superior performance of GOGANPA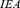 over GOGANPA, GANPA, and absM in our experiments highlights the importance of functional-linkages information, the power of GO IEA annotations, and the usefulness of GO semantic similarity measures. A natural extension of our current development is to consider incorporating gene-network information into a more general GO or pathway enrichment analysis setting, where a set of significantly differentially-expressed genes, or a set of genes of interests, is first identified, and gene-weights are then incorporated into the GO or pathway enrichment tests. Potentially, all the network construction and weight evaluation procedures described in this article can still be used in the GO or pathway enrichment analysis setting, thereby providing biologists an alternative way to analyze gene sets, while accounting for functional linkages between genes.

## Supporting Information

Figure S1
**Goodness-of-fit Measures for the Scale-Free-Topology Criterion.** The goodness-of-fit measure, 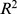, is calculated across a range of thresholds 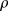. For the GO network constructed without considering electronically curated annotation (No IEA), 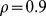 achieves the maximum 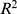, while 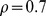 gives the highest 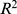 for the network constructed using both manually and electronically curated annotation (With IEA).(PDF)

Table S1
**p53 Data - Further Results.** This table compares the 5 methods discussed in the main article, plus GOGANPA and GOGANPA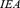 with 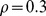 and 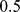, indicated by the subscripts of GOGANPA and GOGANPA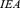. Gene sets with 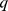-values 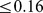 obtained by one of the methods are listed. Number of significant pathways discovered at FDR threshold at 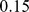 and 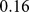 are presented. 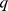-value 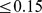 are boldfaced. Abbreviation: GOG: GOGANPA; st dictyostelium: st dictyostelium discoideum camp chemotaxis pathway; rad. sens.: radiation sensitivity; p53 sig.: p53 signalling; st interleukin: st interleukin 4 pathwya; sa trka: Sa trka receptor; bcl2family: bcl2family and regulatory network; dna dam. sig.: DNA damage signalling; st wnt ca2: st wnt Ca2 cyclic GMP pathway; cc: cell cycle; map00910: map00910 nitrogen metabolism. #sig.: number of significant pathways.(PDF)

Table S2
**Breast Cancer Data - Further Results.** Pathways deemed significant at 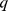-value threshold 0.15, and have 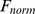 ranked above 80 in all three data sets by at least one method are listed. The rankings of the pathway obtained from the three breast cancer data sets are recorded. Rankings above 80 across all three data sets are boldfaced. GOGANPA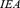 has identified the most number of conserved pathways across the three data sets. Abbreviation: syn. di/tri-phosph.: synthesis and interconversion of nucleotide di and triphosphates; metabolism nts.: metablism of nucleotides; aldo. reg. Na. reabs.: aldosterone regulated sodium reabsorption; APC: regulation of APC/C activators between G1/S and early anaphase; reg. actin cyto.: regulation of actin cytoskeleton; down strm. sig. trans.: down stream signal transduction; CDC20: Cdc20 Phospho-APC/C mediated degradation of Cyclin A. # cons.: number of conserved pathways.(PDF)
