# Supplementary material for: Functional-Network-Based Gene Set Analysis Using Gene-Ontology
Source: PLoS One. 2013 Feb 13;8(2):e55635. doi: 10.1371/journal.pone.0055635 (PMC3572115; doi:10.1371/journal.pone.0055635)
Supplement: Figure S1 — Goodness-of-fit Measures for the Scale-Free-Topology Criterion. The goodness-of-fit measure, , is calculated across a range of thresholds . For the GO network constructed without considering electronically curated annotation (No IEA), achieves the maximum , while gives the highest for the network constructed using both manually and electronically curated annotation (With IEA). (PDF) [file pone.0055635.s001.pdf]

Functional-Network-based Gene Set Analysis using Gene-Ontology: Supplementary Figure S1

Billy Chang, Rafal Kustra, Weidong Tian

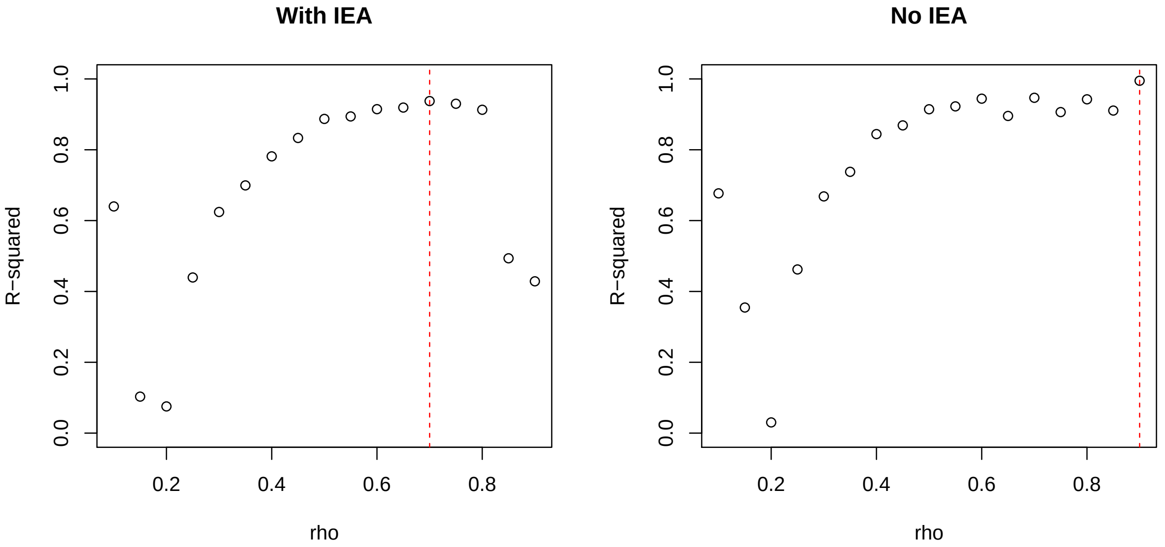

Figure S1. Goodness-of-fit Measures for the Scale-Free-Topology Criterion. The goodness-of-fit measure, R^2, is calculated across a range of thresholds ρ. For the GO network constructed without considering electronically curated annotation (No IEA), $\rho = 0.9$ achieves the maximum R^2, while $\rho = 0.7$ gives the highest R^2 for the network constructed using both manually and electronically curated annotation (With IEA).
