# Supplementary material for: Functional-Network-Based Gene Set Analysis Using Gene-Ontology
Source: PLoS One. 2013 Feb 13;8(2):e55635. doi: 10.1371/journal.pone.0055635 (PMC3572115; doi:10.1371/journal.pone.0055635)
Supplement: Table S1 — p53 Data - Further Results. This table compares the 5 methods discussed in the main article, plus GOGANPA and GOGANPA with and , indicated by the subscripts of GOGANPA and GOGANPA. Gene sets with -values obtained by one of the methods are listed. Number of significant pathways discovered at FDR threshold at and are presented. -value are boldfaced. Abbreviation: GOG: GOGANPA; st dictyostelium: st dictyostelium discoideum camp chemotaxis pathway; rad. sens.: radiation sensitivity; p53 sig.: p53 signalling; st interleukin: st interleukin 4 pathwya; sa trka: Sa trka receptor; bcl2family: bcl2family and regulatory network; dna dam. sig.: DNA damage signalling; st wnt ca2: st wnt Ca2 cyclic GMP pathway; cc: cell cycle; map00910: map00910 nitrogen metabolism. #sig.: number of significant pathways. (PDF) [file pone.0055635.s002.pdf]

Functional-Network-based Gene Set Analysis using Gene-Ontology: Supplementary Table S1

Billy Chang, Rafal Kustra, Weidong Tian

Table S1. p53 Data - Further Results

Pathway	KS	absM	GANPA	$GOG^{IEA}_{0.3}$	$GOG_{0.3}$	$GOG^{IEA}_{0.5}$	$GOG_{0.5}$	$GOG^{IEA}_{0.7}$	$GOG_{0.9}$
p53 hypoxia	**0.001**	**0.015**	**0.01**	**0**	**0.021**	**0.01**	**0.01**	**0.005**	**0.015**
hsp 27	**0.002**	**0.033**	**0.09**	**0.147**	**0.092**	**0.057**	**0.082**	**0.029**	**0.033**
p53	**0.006**	**0.015**	**0**	**0.026**	**0.026**	**0.015**	**0.015**	**0**	**0.01**
p53 up	**0.01**	**0.015**	**0**	**0.021**	**0**	**0**	**0**	**0**	**0**
rad. sens.	**0.064**	**0.015**	**0**	**0.021**	**0.026**	**0.014**	**0.015**	**0**	**0.014**
ck1	0.474	0.178	0.157	0.157	0.209	0.151	0.196	**0.139**	**0.145**
bad	0.507	**0.079**	**0.125**	**0.09**	**0.092**	**0.103**	**0.107**	**0.049**	**0.067**
p53 sig.	0.517	0.22	**0.125**	0.242	0.225	**0.136**	**0.107**	**0.041**	0.209
st dictyostelium	0.788	0.178	0.157	0.157	0.209	**0.136**	**0.107**	**0.106**	**0.145**
g2	0.8	0.22	0.198	0.242	0.225	0.151	**0.137**	**0.106**	0.212
st interleukin 4	0.803	0.223	0.198	0.242	0.225	0.232	0.221	0.156	0.223
sa trka	0.805	0.232	0.313	0.269	0.238	0.281	0.305	0.156	0.234
bcl2family	0.828	0.22	**0.125**	0.242	0.225	0.151	**0.107**	**0.08**	**0.141**
chrebp	0.841	0.22	0.162	0.235	0.225	0.151	**0.133**	0.2	0.209
cr death	0.853	0.223	0.171	0.246	0.263	0.158	**0.145**	0.156	0.202
dna dam. sig.	0.862	0.178	0.198	0.242	0.225	0.203	0.164	0.2	**0.141**
st wnt ca2	0.867	0.22	0.151	0.242	0.225	0.232	0.162	0.203	0.209
ceramide	0.874	0.189	0.157	0.242	0.225	0.151	0.164	**0.038**	0.177
mitochondria	0.881	0.178	**0.127**	0.235	0.217	**0.136**	**0.107**	**0.106**	**0.044**
go ros	0.897	0.232	0.203	0.242	0.225	0.232	0.229	0.156	0.234
cc	0.899	0.178	0.151	0.235	0.209	**0.136**	**0.107**	**0.107**	**0.145**
map00910	0.918	0.178	0.158	0.221	0.225	0.151	0.239	0.2	0.166
cc arrest	0.958	0.22	0.157	0.242	0.225	0.151	**0.107**	**0.095**	0.209
cc regulator	0.969	0.178	**0.125**	0.235	0.209	**0.126**	**0.107**	**0.078**	0.152
il7	1	0.223	0.232	0.242	0.225	0.22	**0.107**	0.203	0.214
#sig.(q-value$\leq$ 0.15)	5	6	10	6	6	11	17	16	12
#sig.(q-value$\leq$ 0.16)	5	6	17	8	6	19	17	20	13

This table compares the 5 methods discussed in the main article, plus GOGANPA and GOGANPA^{IEA} with $\rho = 0.3$ and 0.5, indicated by the subscripts of GOGANPA and GOGANPA^{IEA}. Gene sets with q-values ≤ 0.16 obtained by one of the methods are listed. Number of significant pathways discovered at FDR threshold at 0.15 and 0.16 are presented. q-value ≤ 0.15 are boldfaced. Abbreviation: GOG: GOGANPA; st dictyostelium: st dictyostelium discoideum camp chemotaxis pathway; rad. sens.: radiation sensitivity; p53 sig.: p53 signalling; st interleukin: st interleukin 4 pathwya; sa trka: Sa trka receptor; bcl2family: bcl2family and regulatory network; dna dam. sig.: DNA damage signalling; st wnt ca2: st wnt Ca2 cyclic GMP pathway; cc: cell cycle; map00910: map00910 nitrogen metabolism. #sig.: number of significant pathways.
