# Supplementary material for: Functional-Network-Based Gene Set Analysis Using Gene-Ontology
Source: PLoS One. 2013 Feb 13;8(2):e55635. doi: 10.1371/journal.pone.0055635 (PMC3572115; doi:10.1371/journal.pone.0055635)
Supplement: Table S2 — Breast Cancer Data - Further Results. Pathways deemed significant at -value threshold 0.15, and have ranked above 80 in all three data sets by at least one method are listed. The rankings of the pathway obtained from the three breast cancer data sets are recorded. Rankings above 80 across all three data sets are boldfaced. GOGANPA has identified the most number of conserved pathways across the three data sets. Abbreviation: syn. di/tri-phosph.: synthesis and interconversion of nucleotide di and triphosphates; metabolism nts.: metablism of nucleotides; aldo. reg. Na. reabs.: aldosterone regulated sodium reabsorption; APC: regulation of APC/C activators between G1/S and early anaphase; reg. actin cyto.: regulation of actin cytoskeleton; down strm. sig. trans.: down stream signal transduction; CDC20: Cdc20 Phospho-APC/C mediated degradation of Cyclin A. # cons.: number of conserved pathways. (PDF) [file pone.0055635.s003.pdf]

Functional-Network-based Gene Set Analysis using Gene-Ontology: Supplementary Table S2

Billy Chang, Rafal Kustra, Weidong Tian

Table S2. Breast Cancer Data - Further Results

Pathway	absM	GANPA	$GOG^{IEA}_{0.3}$	$GOG_{0.3}$	$GOG^{IEA}_{0.5}$	$GOG_{0.5}$	$GOG^{IEA}_{0.7}$	$GOG_{0.9}$
syn. di/tri-phosph.	**1,23,7**	**4,12,14**	**1,25,7**	**2,22,6**	**1,22,5**	**1,18,5**	**1,27,6**	**1,24,7**
metablism nts	4,81,6	**6,54,7**	**2,72,5**	**3,64,4**	**2,70,4**	**2,63,3**	**3,56,10**	**4,78,10**
focal adhesion	**8,25,54**	**12,29,59**	**8,23,78**	**8,24,70**	8,25,85	**8,23,46**	**5,24,37**	**6,21,48**
pathways in cancer	**14,17,37**	**14,18,30**	**15,18,35**	**17,21,31**	**16,19,33**	**19,20,36**	**21,18,24**	**19,19,40**
AGR	**20,18,1**	**33,30,1**	**13,12,1**	**30,29,2**	**15,23,1**	**21,34,2**	**37,80,1**	**20,18,1**
melanoma	27,152,101	25,96,77	26,156,93	46,184,109	20,129,90	40,158,114	**17,69,35**	16,115,80
acute myeloid leukemia	**28,28,57**	**47,42,62**	**19,26,48**	**32,28,59**	**41,36,70**	49,42,111	**27,30,18**	**26,29,60**
pancreatic cancer	30,39,85	**34,36,48**	18,37,86	21,33,82	**28,56,58**	**34,78,53**	**36,67,39**	**34,39,76**
G2/M transition	38,30,90	**31,32,58**	41,38,84	35,30,81	**39,33,78**	37,27,81	34,15,108	36,30,89
prostate cancer	**39,12,12**	**37,19,8**	**33,9,10**	**49,14,13**	**46,14,7**	**54,22,9**	**62,19,2**	**45,5,9**
p53 signaling	**40,9,24**	**30,5,60**	**43,10,26**	**42,5,48**	**40,4,39**	**58,8,63**	33,6,88	**39,9,22**
hemostasis	41,100,50	56,102,45	37,100,60	40,100,60	37,97,49	**11,77,40**	28,95,48	37,90,36
axon guidance	**48,8,11**	**61,9,4**	**61,4,14**	**53,4,9**	**55,16,8**	**72,17,11**	**51,8,5**	**50,10,11**
PDGF	50,96,114	21,58,81	59,95,117	55,93,128	42,88,80	47,114,107	**25,40,47**	64,114,125
cell cycle	**55,22,80**	35,17,106	53,24,83	50,17,84	**52,30,68**	**56,29,59**	44,7,128	55,20,86
renal cell carcinoma	**71,55,10**	93,55,10	**75,52,29**	**76,69,29**	**61,72,31**	87,94,22	**60,72,12**	85,56,8
aldo. reg. Na reabs.	76,163,124	90,77,109	68,194,153	67,198,146	66,161,190	63,97,132	**58,78,71**	90,171,130
APC	**80,53,22**	**65,48,18**	**77,66,19**	**72,57,17**	**72,55,15**	**64,53,14**	**30,9,57**	82,51,20
reg. actin cyto.	84,87,71	77,61,84	70,77,101	82,98,90	70,86,108	70,80,99	65,90,53	**75,71,59**
down strm. sig. trans.	87,102,53	**53,60,54**	91,96,42	90,92,58	87,92,45	96,98,64	**56,51,25**	70,88,39
cell surface	90,58,73	180,80,69	85,67,76	**80,68,74**	**79,58,64**	**62,31,34**	113,107,62	92,58,71
cdc20	92,113,15	81,111,12	94,120,13	92,111,11	89,110,12	75,103,10	**43,47,33**	91,111,15
longevity	109,154,87	73,112,55	159,147,123	166,141,115	111,148,56	114,152,37	**48,76,72**	108,149,87
glioma	111,151,77	**74,64,37**	119,141,85	132,139,77	112,115,60	103,116,58	83,98,27	104,144,66
# cons.	11	14	11	12	14	13	17	11

Pathways deemed significant at q-value threshold 0.15, and have F_{norm} ranked above 80 in all three data sets by at least one method are listed. The rankings of the pathway obtained from the three breast cancer data sets are recorded. Rankings above 80 across all three data sets are boldfaced. $GOGANPA^{IEA}$ has identified the most number of conserved pathways across the three data sets. Abbreviation: syn. di/tri-phosph.: synthesis and interconversion of nucleotide di and triphosphates; metabolism nts.: metablism of nucleotides; aldo. reg. Na. reabs.: aldosterone regulated sodium reabsorption; APC: regulation of APC/C activators between G1/S and early anaphase; reg. actin cyto.: regulation of actin cytoskeleton; down strm. sig. trans.: down stream signal transduction; CDC20: Cdc20 Phospho-APC/C mediated degradation of Cyclin A. # cons.: number of conserved pathways.
